# Food and water security issues in Russia I: food security in the general population of the Russian Arctic, Siberia and the Far East, 2000–2011

**DOI:** 10.3402/ijch.v72i0.21848

**Published:** 2013-10-23

**Authors:** Alexey A. Dudarev, Pavel R. Alloyarov, Valery S. Chupakhin, Eugenia V. Dushkina, Yuliya N. Sladkova, Vitaliy M. Dorofeyev, Tatijana A. Kolesnikova, Kirill B. Fridman, Lena Maria Nilsson, Birgitta Evengård

**Affiliations:** 1 Northwest Public Health Research Center, St. Petersburg, Russia; 2 Dubna City Hospital, Moscow oblast, Russia; 3 Division of Nutritional Research, Department of Public Health and Clinical Medicine, Umeå University, Umeå, Sweden; 4 Arcum – Arctic Research Centre, Umeå University, Umeå, Sweden; 5 Division of Infectious Diseases, Department of Clinical Microbiology, Umeå University, Umeå, Sweden

**Keywords:** food security, chemical, biological contamination, Russian Arctic

## Abstract

**Background:**

Problems related to food security in Russian Arctic (dietary imbalance, predominance of carbohydrates, shortage of milk products, vegetables and fruits, deficit of vitamins and microelements, chemical, infectious and parasitic food contamination) have been defined in the literature. But no standard protocol of food security assessment has been used in the majority of studies.

**Objectives:**

Our aim was to obtain food security indicators, identified within an Arctic collaboration, for selected regions of the Russian Arctic, Siberia and the Far East, and to compare food safety in these territories.

**Study design and methods:**

In 18 regions of the Russian Arctic, Siberia and the Far East, the following indicators of food security were analyzed: food costs, food consumption, and chemical and biological food contamination for the period 2000–2011.

**Results:**

Food costs in the regions are high, comprising 23–43% of household income. Only 4 out of 10 food groups (fish products, cereals, sugar, plant oil) are consumed in sufficient amounts. The consumption of milk products, eggs, vegetables, potatoes, fruits (and berries) is severely low in a majority of the selected regions. There are high levels of biological contamination of food in many regions. The biological and chemical contamination situation is alarming, especially in Chukotka. Only 7 food pollutants are under regular control; among pesticides, only DDT. Evenki AO and Magadan Oblast have reached peak values in food contaminants compared with other regions. Mercury in local fish has not been analyzed in the majority of the regions. In 3 regions, no monitoring of DDT occurs. Aflatoxins have not been analyzed in 5 regions. Nitrates had the highest percentage in excess of the hygienic threshold in all regions. Excesses of other pollutants in different regions were episodic and as a rule not high.

**Conclusion:**

Improvement of the food supply and food accessibility in the regions of the Russian Arctic, Siberia and the Far East is of utmost importance. Both quantitative and qualitative control of chemical and biological contaminants in food is insufficient and demands radical enhancement aimed at improving food security.

Food security, defined as a situation “when all people, at all times, have physical and economic access to sufficient, safe and nutritious food to meet their dietary needs and food preferences for an active and healthy life” (1), which is an urgent issue worldwide. Climate change and chemical contaminants have been shown to endanger food security, especially in the vulnerable ecosystem of the Arctic (2, 3). Consequently, as a prioritized project within the Arctic Council, indicators of food security have been identified recently in a collaboration between the Sustainable Development Work Group/Arctic Human Health Expert Group (SDWG/AHHEG) and the Arctic Monitoring and Assessment Programme (AMAP) Human Health Assessment Group (4).

In Russia, several problems related to food security in the Arctic have been identified previously. Among the general population, the polar tension syndrome^1^ has been observed (5), as have problems regarding a shift of macronutrients in the diet towards carbohydrates (an abundance of sugar, confectioneries, bread, pasta, cereals), shortages of milk, milk products, vegetables and fruits, and, therefore, a lack of almost all types of vitamins, mineral nutrients (particularly calcium, phosphorus, magnesium, potassium, iodine, zinc, fluorine, etc.), and contamination of food (mainly local) by pesticides, metals, antibiotics, nitrates and biological agents (6).

Dietary imbalances and malnutrition have been ascertained in Murmansk city (7), Arkhangelsk city (8), Yamalo-Nenets AO (9), Krasnoyarsk Kraj (10), Yakutia (Sakha) Republic (11), Primorsky Kraj (12, 13) and Khabarovsk Kraj (14). A general lack of vitamins and mineral nutrients in the diet has been recorded in Komi Republic (15), Yamalo-Nenets AO (16, 17), Yakutia/Sakha Republic (18) and Magadan Oblast (19). A deficit of selenium in the diet was noted in Khanty-Mansi AO (20), Magadan Oblast, Sakhalin Oblast, Kamchatka Kraj, Khabarovsk Kraj (21) and Yakytia (22). A deficit of iodine in the diet was noted in Arkhangelsk Oblast (23, 24), Krasnoyarsk Kraj (25), Yakutia, Sakhalin Oblast, Kamchatka Kraj, Khabarovsk Kraj (26, 27) and Primorsky Kraj (28).

Microparasitic diseases of humans and animals (helminthiases) have been recorded generally in Russian Northern regions (29); parasite contamination of fish in Murmansk Oblast (30), Yamalo-Nenets AO (31), Yakutia (32), Kamchatka (33) and Chukotka (34, 35); microbial contamination of fish in Primorsky Kraj (36), microbial and chemical contamination of fish in Khabarovsk Kraj (37, 38).

Contamination of food products (bread, vegetables, milk products) by metals (arsenic, lead, mercury and cadmium) was noted in Krasnoyarsk Kraj (10).

As a part of the previously mentioned Arctic collaboration project on food security, a literature search based on Russian scientific peer-reviewed journals (mainly 2000–2012) was performed. This literature search did not reveal unified indicators of food security for comparative assessment of selected Russian regions because the majority of studies diverged widely; no standard protocol of food security assessment had been used. As a rule, studies were narrowly aimed, small in scope, regionally self-contained, and focused on specific food problems in specific regions. In addition, some of the indicators readily available in statistical sources of Russia were not promoted in the international collaboration because of lack of comparable international data (4). Thus, it is an urgent task to present food security indicators from the northernmost regions of Russia in a country-specific report.

This study is the first complex comparative assessment of food security in the regions of the Russian Arctic, Siberia and the Far East using unified food security indicators collected from statistical sources.

## Objectives

The general aim was to obtain food security indicators, identified within the international collaboration project on food security (4), for the selected regions of the Russian Arctic, Siberia and the Far East (for the period 2000–2011), and to compare food safety in these territories.

## Study design and methods

**Fig. 1:**
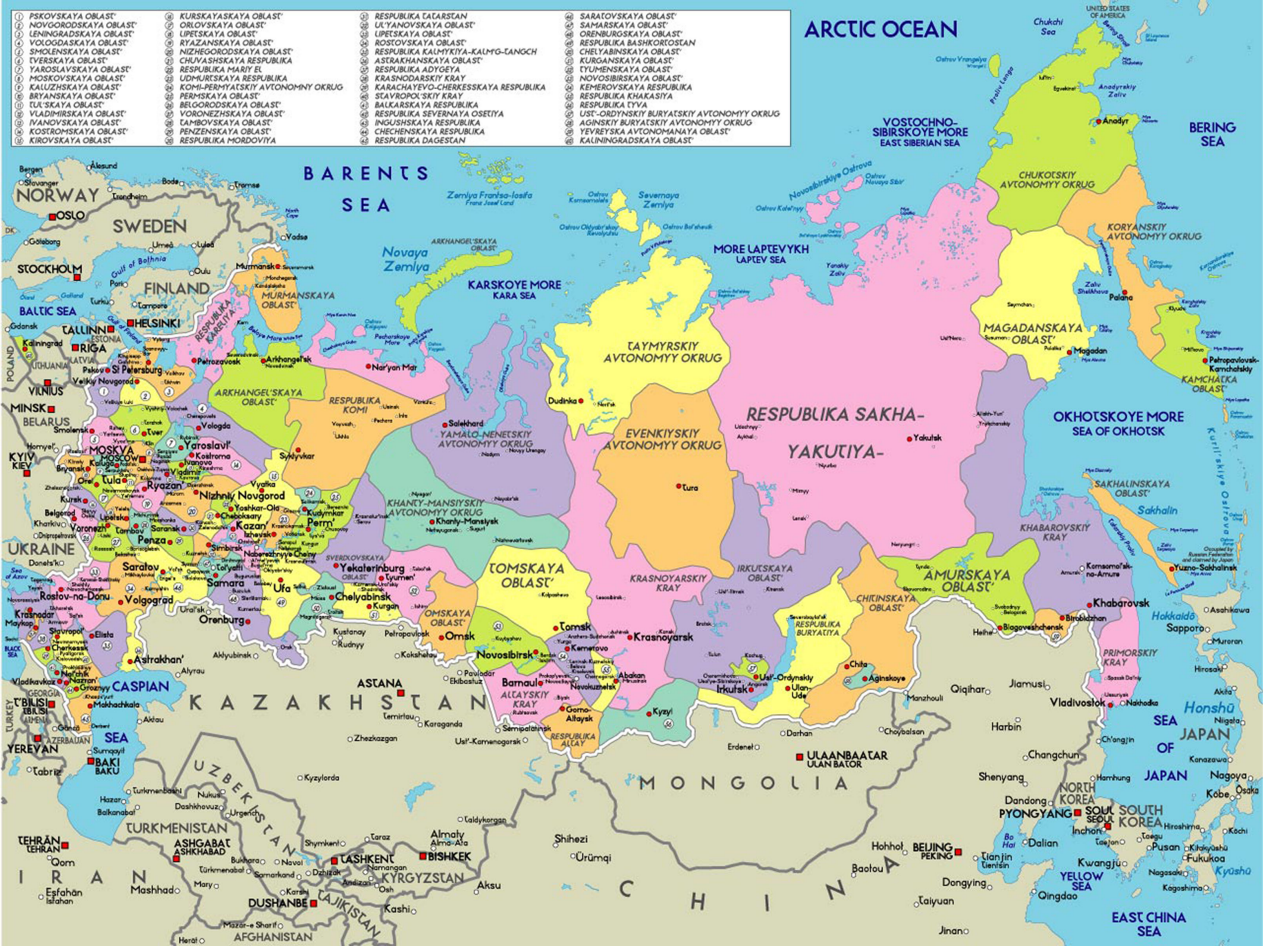
Administrative division of Russia.

Eighteen regions of the Russian north, Siberia and the Far East (Fig. 1) have been included in the study (from west to east): Murmansk Oblast, Karelia Republic, Arkhangelsk Oblast, Nenets AO (Autonomous Okrug), Komi Republic, Yamalo-Nenets AO, Khanty-Mansi AO, Taymyr AO, Evenki AO, Sakha Republic, Magadan Oblast, Koryak AO, Chukotka AO, Kamchatka Oblast, Sakhalin Oblast, Khabarovsk Kraj, Primorsky Kraj and Amur Oblast.

Accessible (open) official statistical data on food security for these 18 regions (Oblast, republic, Autonomous Okrug, Kraj) were collected (for 2000–2011) from 3 sources of information:

- Regional Statistical Yearbooks (trade statistics) – all regions except Khanty-Mansi AO, Taymyr AO, Evenki AO, Koryak AO and Sakhalin Oblast.
- Regional State Reports on the “Sanitary-Epidemiological Situation” (excesses in percentages of national hygienic limits of chemical and biological food contamination) – all regions except Taymyr AO, Evenki AO, Koryak AO and Primorsky Kraj.
- The Federal Automatic system “Social-Hygienic Monitoring” (data on chemical pollution of different types of food products) – all regions except Koryak AO.

The first 2 types of regional documents became accessible for the public only in the mid-2000s, and they cover the first decade of the millennium. The Federal Automatic system was launched recently, and it is now developing; its quality is not good enough yet. Data on food, water, soil, air sample analyses (as well as cases of infectious and parasitic diseases) in all regions of the Russian Federation during the last years are accumulating in the system with the aim of further monitoring. This registry was formed on the basis of Federal Center of Hygiene and Epidemiology of Rospotrebnadzor – Federal Service of Oversight on Protection of the Rights of Consumers and Human Well-Being (formerly the Sanitary-Epidemiological Surveillance Service); the registry has a restricted Website (special permission is needed to get access). Data are presented in an Excel format without systematization; its content has no description in documents or reports.

As for statistics on chemical contamination of local traditional food consumed by indigenous people, the information (on metals and organochlorines) is available only from the Russian Arctic PTS (persistent toxic substances) study of 2001–2004 for 4 regions: Murmansk Oblast, Nenets AO, Taymyr AO and Chukotka AO (39, 40). Nevertheless, some data on chemical contamination of locally produced food compared to imported food (from other Russian regions and from outside Russia) were collected from the Federal Automatic system “Social-Hygienic Monitoring” for each selected region.

The following available (in official statistics) indicators of food security have been analyzed in the selected regions:

- Food costs (based on trade statistics and presented as a proportion of household income, %);
- Food consumption (based on trade statistics, 10 food groups, including fruits and berries, presented as a proportion of recommended quantities, %);
- Chemical contaminants in food (including pesticides and mercury);
- Biological contaminants in food (including bacteria, fungi and parasites).

## Results

**Fig. 2:**
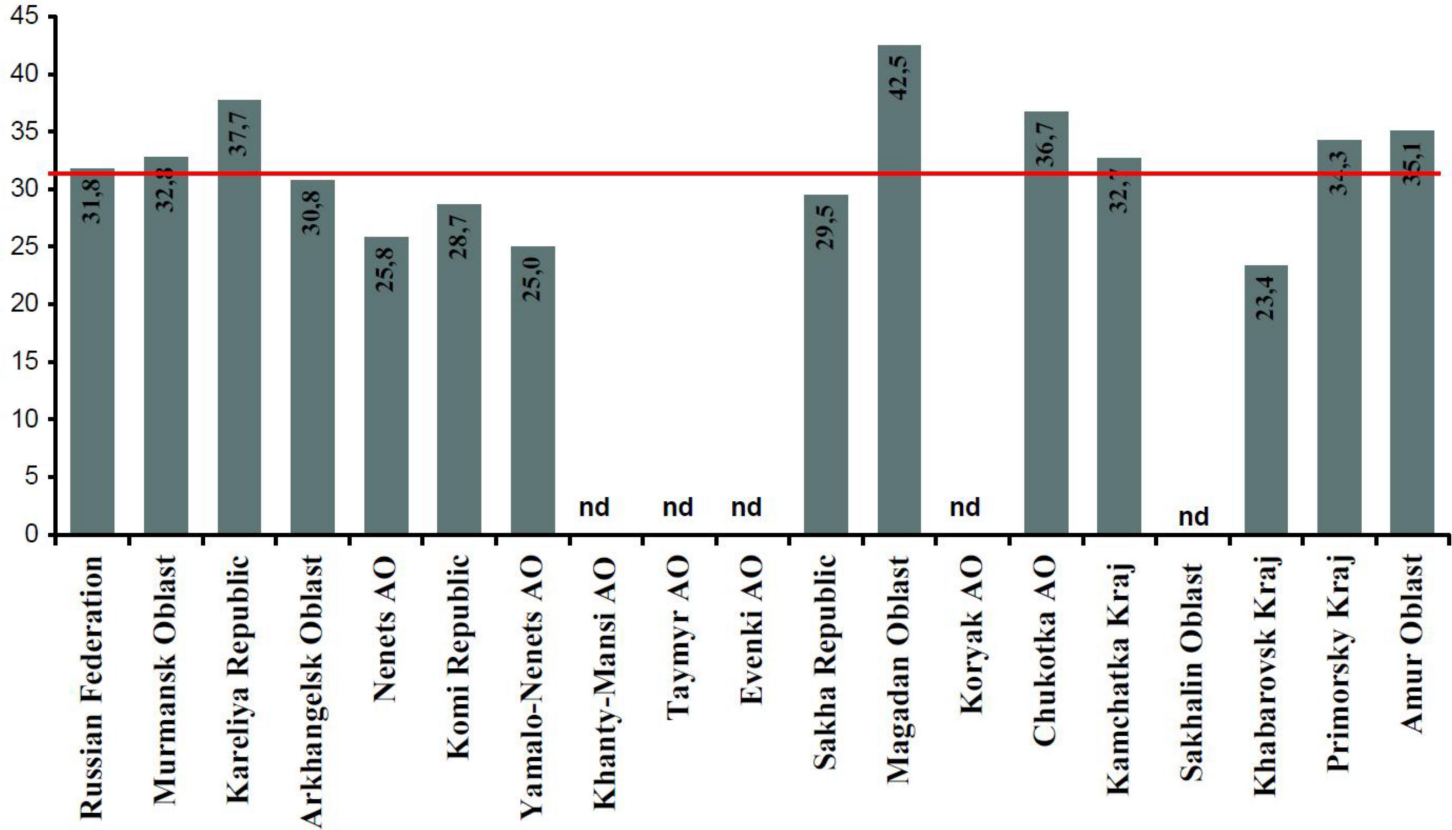
Food costs as proportion of household income, averaged 2000–2010, %.

Food costs as a proportion of household income [data from national and regional Statistical Yearbooks (41, 42)] are presented in Fig. 2. Generally, food costs in Russia are high – they average 31.8% of household income. In the selected regions, the indices fluctuate from 23 to 43%. The highest values were reported from Karelia, Magadan Oblast and Chukotka; the lowest, from Nenents AO, Yamalo-Nenets AO and Khabarovsk Kraj.

The recommended quantities of foods for consumption are determined in Russia by the federal document (43) elaborated by the Ministry of Health (Table I). The actual consumption of 10 groups of products (as a percentage of recommended quantities) in the regions (40–57) is presented in Table II.

**Table I:** Recommended quantities of food consumption in Russia (43)

Product	Unit (kg/person/year)
Meat and meat products	70–75
Milk and milk products	320–340
Fish and fish products	18–22
Cereals (including grains, bread, macaroni, beans)	95–105
Eggs (pieces)	260 pieces
Sugar	24–28
Plant oil	10–12
Vegetables	120–140
Potatoes	95–100
Fruits and berries	90–100

**Table II:** Food consumption in the selected regions, percentage of recommended quantities

	Years	Meat*	Milk*	Fish*	Cereals*	Eggs (pieces)	Sugar	Plant oil	Vegetables	Potatoes	Fruits, berries
Russian Federation	2009	87	77	83	113	101	132	109	86	113	62
Murmansk Oblast	2000–08	67	56	100	100	79	120	108	63	71	48
Karelia Republic**	2008–10	79	87	99	51	78	94	67	nd	84	nd
Arkhangelsk Oblast**	2002–10	63	46	132	102	78	129	100	61	88	55
Nenets AO	nd	nd	nd	nd	nd	nd	nd	nd	nd	nd	nd
Komi Republic	nd	nd	nd	nd	nd	nd	nd	nd	nd	nd	nd
Yamalo-Nenets AO	2000–10	118	69	100	92	77	98	100	76	62	75
Khanty-Mansi AO	nd	nd	nd	nd	nd	nd	nd	nd	nd	nd	nd
Taymyr AO	nd	nd	nd	nd	nd	nd	nd	nd	nd	nd	nd
Evenki AO	nd	nd	nd	nd	nd	nd	nd	nd	nd	nd	nd
Yakutia/Sakha Republic	2008	104	82	93	103	65	100	96	40	49	56
Magadan Oblast	2000–08	96	49	107	99	62	100	nd	63	68	53
Koryak AO	nd	nd	nd	nd	nd	nd	nd	nd	nd	nd	nd
Chukotka AO	2008	100	58	80	87	54	95	60	50	29	51
Kamchatka Kraj	2003–08	105	61	120	96	70	100	100	65	84	62
Sakhalin Oblast	2008	115	63	120	101	69	116	120	74	75	64
Khabarovsk Kraj**	2002–07	88	63	105	100	74	100	107	77	82	55
Primorsky Kraj	2000–10	66	33	106	92	62	124	100	82	120	52
Amur Oblast**	2009–10	87	59	109	92	107	100	108	49	nd	57

* Meat and meat products, milk and milk products, fish and fish products, cereals (including grains, bread, macaroni, beans).

** Data were recalculated (from kg/person/year to percentage of recommended quantities) from Regional Statistical Yearbooks (41, 42) or from **Regional Reports on the “Sanitary-Epidemiological Situation” (49, 50) and have been averaged for the specified periods.

nd =no data.

Out of 10 groups of products, only 4 groups – fish (and fish products), cereals (including grains, bread, macaroni, beans), sugar and plant oil – are consumed in sufficient amounts by the population of the selected regions. While on average the consumption in Russia of fish (and fish products) constitutes 83% of the recommended level, in a majority of the selected regions these values tend to rise to 100% and more, except for Chukotka. Cereal consumption is characterized by high quantities (87–102%) with the exception of Karelia (51%). Sugar is consumed everywhere rather actively – 93–130%. Plant oil consumption is close to recommended quantities in all regions with the exception of Karelia (67%) and Chukotka (60%).

Meat consumption is very different in the regions; while Murmansk Oblast, Arkhangelsk Oblast and Primorsky Kraj are defined as areas with low meat consumption (<70%), the people of Yamalo-Nenets AO, Yakutia/Sakha, Magadan Oblast, Chukotka, Kamchatka and Sakhalin are high meat consumers (95–118%).

A very low consumption of milk (and milk products), eggs, vegetables, potatoes, fruits (and berries) is obvious in a majority of the selected regions. More than 80% of the recommended quantities of milk and milk products are consumed only in Karelia and Yakutia, while in Arkhangels Oblast, Magadan Oblast and Primorsky Kraj the consumption of this type of products constitutes less than 50% of the recommended quantities. Egg intake is less than 80% everywhere except Amur Oblast. Vegetable consumption is very low (<50%) in Yakutia, Chukotka and Amur Oblast.

Intake of fruits and berries is particularly alarming in the majority of the regions; only in Yamalo-Nenets AO is the consumption of these vitamin-supplying products at about 75%, while in other regions this value is 48–64%. It is worth underlining that the average level of fruit consumption in Russia is also low: 62%. Recommended quantities of fruits and berries (90–100 kg/person/year) correspond to 247–274 g/day/person, but actual consumption averages about 130 g/day/person in the selected regions. Therefore, hypovitaminosis may occur in the population.

## Russian hygienic regulations of food contamination

The Sanitary Rules and Norms “Hygienic Safety and Nutritional Value of Food Products” (44) with multiple amendments is the main document in Russia regulating safety of food (of both animal and plant origin) in terms of chemical, biological and radioactive contamination of all types of existing food and food raw materials; it is the Russian analogue of Codex Alimentarius. There are important additional documents on regulations of pesticide content in environmental objects (including food) (45), on requirements for food additive use (46), requirements for baby food (47) and on chemicals released from materials in contact with food (48).

The biological safety of foods is regulated by the main standard (44) and includes the following groups of agents:

- Microbial pathogens and agents of parasitic diseases and toxins that cause infectious and parasitic diseases hazardous to human and animal health are not allowed;
- Mesophilic, aerobic and facultative–anaerobic coliform bacteria of the family Enterobacteriaceae, enterococci are not allowed;
- Conditionally pathogenic microorganisms, which include: *E. coli*, *S. aureus*, bacteria genus Proteus, *B. cereus* and sulfite-reducing clostridia, and *Vibrio parahaemolyticus*, are not allowed;
- Pathogenic microorganisms including *Salmonella* and *Listeria monocytogenes*, bacteria of the genus Yersinia are not allowed;
- Spoilage microorganisms (yeasts and moulds, lactic acid bacteria) – the standard reflects the number of colony-forming units in 1 g (ml) of the product (CFU /g, ml);
- In products with controlled levels of biotechnological microflora and probiotic products (fermentation microflora and probiotic microorganisms – lactic acid bacteria, propionate bacteria, yeast, bifidobacteria, acidophilus bacteria, etc.). The standard reflects the number of colony-forming units in 1 g (ml) of the product (CFU/g, ml);
- In canned food, microorganisms capable of growing at storage temperature and microorganisms and microbial toxins that are dangerous to human health are not allowed.

The comparative percentages of all food samples in the selected regions which do not obey hygienic norms on biological and chemical food contamination based on regional sanitary-epidemiological reports (49, 50) are presented in Table III.

**Table III:** Food samples analyzed for chemical and biological contaminants in all food and in imported food products in the selected regions, averaged during a specified period, percentage of samples which do not follow hygienic norms

		Chemical contaminants		Biological contaminants	
			
	Years	All food	Imported food	All food	Imported food
Russian Federation	2000–10	3.7	2.3	5.9	3.4
Murmansk Oblast	2007–11	5.3	nd	5.1	nd
Karelia Republic	2002–11	5.0	nd	7.7	nd
Arkhangelsk Oblast	2002–11	6.8	5.9	11.8	6.2
Nenets AO	2007–11	7.6	1.3	15.3	nd
Komi Republic	2002–11	2.5	3.7	7.9	5.0
Yamalo-Nenets AO	2007–11	5.5	nd	7.6	5.5
Khanty-Mansi AO	2010–11	2.5	3.4	10.9	6.1
Taymyr AO	2006–11	1.4	nd	nd	nd
Evenki AO	2006–11	4.8	nd	nd	nd
Sakha Republic	2002–11	5.7	3.6	12.8	6.0
Magadan Oblast	2002–10	5.3	3.7	11.1	9.1
Koryak AO	nd	nd	nd	nd	nd
Chukotka AO	2007–11	13.2	19.7	14.4	5.5
Kamchatka Kraj	2008–11	1.9	5.9	8.6	2.4
Sakhalin Oblast	2005–11	5.3	3.3	10.8	6.1
Khabarovsk Kraj*	2003–11	2.7	6.7	8.1	4.6
Primorsky Kraj	nd	nd	nd	nd	nd
Amur Oblast	2005–11	0.5	1.7	6.1	3.1

Data from Refs. 48 and 49.

* For biological contaminants 2004–2011 time period was averaged.

nd=no data.

The highest levels of biological contamination of food (8–15%) are registered in Arkhangelsk Oblast, Nenets AO, Khanty-Mansi AO, Sakha Republic, Magadan Oblast, Chukotka AO and Sakhalin Oblast. These levels are also higher than the average level in Russia (6%). Chemical contamination generally was about twice as low and did not fluctuate much (2–7%) in most of regions except for high levels in Chukotka (13%) and low levels in Primorsky Kraj (0.5%). Thus, Chukotka food appears to be highest in terms of both biological and chemical contamination, although Nenets AO has a higher number of biological contaminants (Table III).

Biological contamination of imported food (Table III) is less than all of the food (2.5–9%), but chemical contamination of imported food is similar to that of all food, and in some regions even higher (again in Chukotka the level is almost 20% compared to 13% for all food). Because the Federal Automatic system “Social-Hygienic Monitoring” does not have information on biological food contaminants and other specified documents do not contain data on individual biological contaminants in food, evaluation of the structure of biological food contamination in the selected regions is not possible.

## Chemical contaminants in food

**Fig. 3:**
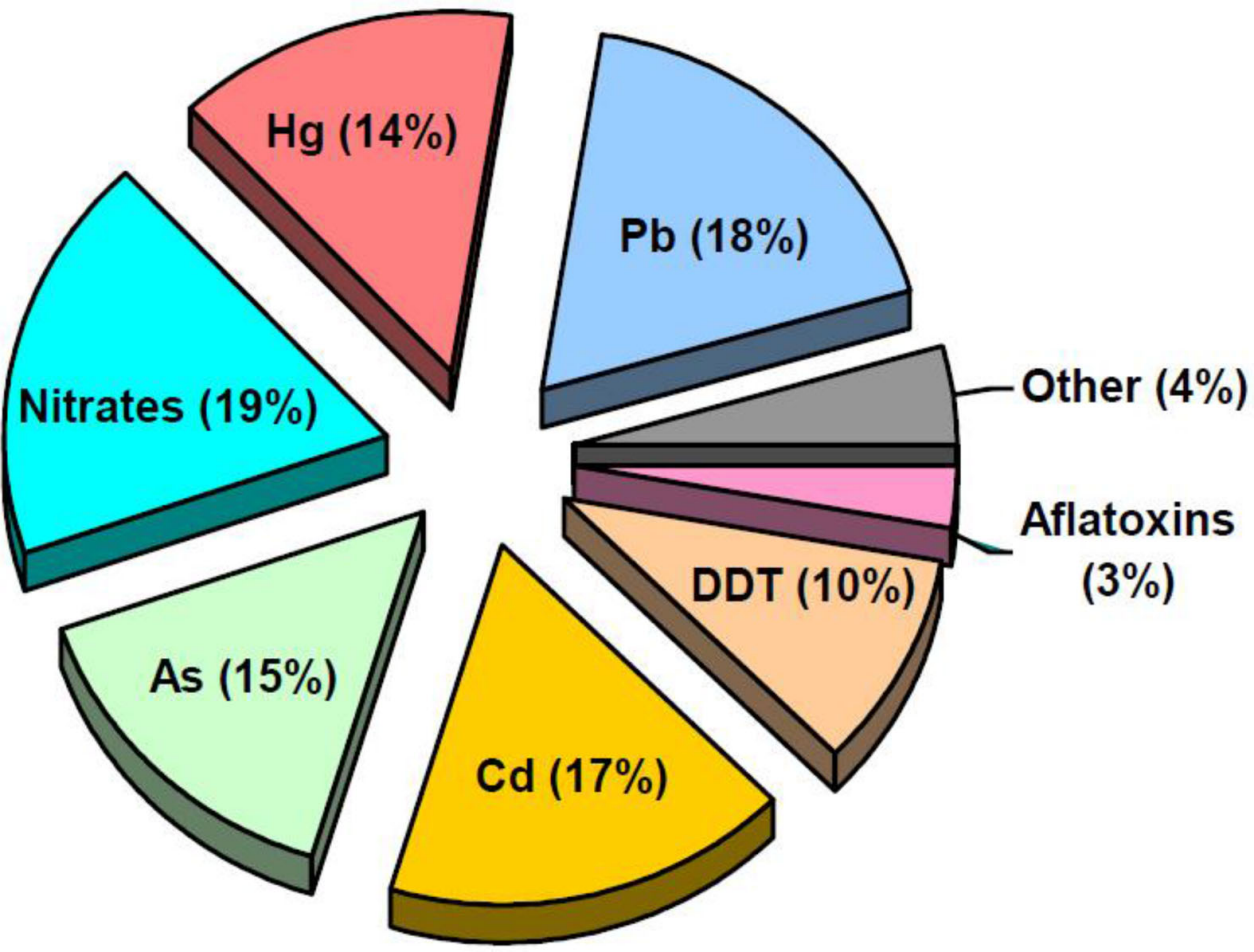
Structure of chemical pollutants in all food samples from all selected regions (2007–11), percentage of total number of samples analyzed.

Collection of data from the Federal Automatic System “Social-Hygienic Monitoring” is estimated at about 178,000 analyzed food samples from all selected regions during 2007–2011. This database has enabled us to evaluate selected contaminants, which are controlled in the regions. The structure of chemical pollutants in this totality is presented in Fig. 3.

Ninety-four percent of food samples have been analyzed on 7 pollutants: 4 metals (lead, cadmium, arsenic, mercury), 1 product of mineral fertilizers (nitrates), 1 pesticide (DDT) and 1 mycotoxin (aflatoxin). Among others (4%) are 17 pollutants: benzo(a)pyrene (0.18%), NDMA/NDEA (1.42%), histamine (0.11%), nitrites (0.01%); 4 mycotoxins: dezoxynilvalenol (0.74%), zearalenone (0.65%), patulin (0.08%) an T-2 toxin (0.56%); 6 metals: iron (0.01%), copper (0.19%), nickel (0.006%), tin (0.09%), zinc (0.16%) and chromium (0.002%); and 3 organochlorines: HCB (0.006%), HCH (0.06%) and PCB (0.06%).

According to the main hygienic standard (46), the control of many contaminants (e.g. dioxins) in food is pursued only in cases of “reasonable assumptions about their possible presence in food.” Thus, of the large number of potential food pollutants, only 7 are under real regular control; among pesticides, only DDT.

Tables IV and V present the numbers of food samples analyzed for all chemical pollutants and 7 separate main pollutants in each selected region averaged during a specified period, and the percentage of samples which exceeded the Russian hygienic threshold.

**Table IV:** Number of food samples analyzed for all chemical pollutants and for nitrates, aflatoxins and DDT in each selected region averaged during a specified period, and percentage of samples which do not obey hygienic norms, separately

		All pollutants		Nitrates		Aflatoxins		DDT		DDT (local)	
						
	Years	n, per 10,000	n, population	n	%	n	%	n	%	n	%
Murmansk Oblast	2007–11	17.7	1,466	634	8	28	0	66	0	34	0
Karelia Republic	2007–11	33.2	2,239	771	2.4	60	0	146	0	95	0
Arkhangelsk Oblast	2007–11	20.5	2,563	778	6.6	51	1.2	229	0.6	150	0.9
Nenets AO	2009–11	21.4	90	76	14.4	ns	ns	2	0	4	0
Komi Republic	2007–11	27.2	2,564	296	5.1	111	1.1	280	0.8	164	0.5
Yamalo-Nenets AO	2007–11	10.2	552	103	11.4	1	0	35	1.7	11	0
Khanty-Mansi AO	2007–11	30.6	4,667	1,374	5	105	3	169	5.1	58	2.1
Taymyr AO	2006–11	56.6	220	51	2	ns	ns	ns	ns	ns	ns
Evenki AO	2006–11	292.2	552	42	2.4	ns	ns	ns	ns	ns	ns
Sakha Republic	2007–11	10.5	998	143	8.5	18	0	66	0.9	32	1.5
Magadan Oblast	2007–11	156.1	2,523	84	13.1	ns	ns	398	0	202	0
Koryak AO	nd	nd	nd	nd	nd	nd	nd	nd	nd	nd	nd
Chukotka AO	2008–11	28.5	142	37	14.4	ns	ns	ns	ns	ns	ns
Kamchatka Kraj	2007–11	24.2	816	85	3.5	42	12.9	105	0.6	127	0.5
Sakhalin Oblast	2009–11	18	919	183	11.1	6	0	69	9.2	36	15.5
Khabarovsk Kraj	2007–11	22.2	3,074	432	11.1	62	0	242	0.7	138	0.9
Primorsky Kraj	2007–11	55.5	10,982	1,237	3.4	451	1.8	1,373	4.7	594	5.3
Amur Oblast	2007–11	17	1,453	285	1	49	0.4	294	0	162	0

Data from “Social–Hygienic Monitoring” system.

n=average number of samples per year.

ns=no samples analyzed.

nd=no data.

%=of samples exceeded threshold.

Total numbers of food samples analyzed in the selected regions (Table IV) are very different (from 90 to 11,000 average/year). Because of this, they have been adjusted to a standard of 10,000 samples to make these indices become more similar (from 10 to 55 per 10,000/year) with the exceptions of Evenki AO (292) and Magadan Oblast (156), which are the peaks in food contaminants laboratory control among the regions.

In looking at 7 main pollutants in food samples (Tables IV and V), we can state that nitrates, lead, cadmium and arsenic are assessed in all selected regions; mercury in local fish has not been analyzed in all regions except Khanty-Mansi AO, Yakytia and Sakhalin Oblast, while mercury is not under control at all in Evenki AO; DDT is out of control in Taymyr AO, Evenki AO and Chukotka. In the last regions and additionally in Nenets AO and Magadan Oblast, aflatoxins also have not been analyzed.

**Table V:** Number of food samples analyzed for metals in each selected region averagely during specified period, and percentage of samples which do not obey hygienic norms

		Pb		Cd		As		Hg		Hg (local fish)	
						
	Years	n	%	n	%	n	%	n	%	n	%
Murmansk Oblast	2007–11	179	0.9	176	0.7	147	0.9	158	0.5	ns	ns
Karelia Republic	2007–11	344	0.2	342	0.1	280	0.5	257	0.4	ns	ns
Arkhangelsk Oblast	2007–11	418	0.4	407	0.3	314	1	311	1	ns	ns
Nenets AO	2009–11	3	0	2	0	3	0	3	0	ns	ns
Komi Republic	2007–11	506	0.4	514	1.8	364	0.4	363	1.1	ns	ns
Yamalo-Nenets AO	2007–11	120	0.7	116	0.5	76	1.3	69	1.7	ns	ns
Khanty-Mansi AO	2007–11	775	2.8	751	0.5	730	0.8	697	0.5	14	2.3
Taymyr AO	2006–11	45	0	45	0	46	0	35	0	ns	ns
Evenki AO	2006–11	160	0	160	0	191	0	ns	ns	ns	ns
Sakha Republic	2007–11	234	2	231	1	140	2.9	149	1.3	11	9.3
Magadan Oblast	2007–11	493	0.1	494	0.4	489	0.4	555	0	ns	ns
Koryak AO	nd	nd	nd	nd	nd	nd	nd	nd	nd	nd	nd
Chukotka AO	2008–11	39	1.9	38	0.7	4	0	24	0	ns	ns
Kamchatka Kraj	2007–11	178	0.1	168	0.1	99	0	98	2	ns	ns
Sakhalin Oblast	2009–11	165	0.8	166	0.4	155	2.8	160	4	3	0
Khabarovsk Kraj	2007–11	770	0.1	510	0.3	454	0.6	461	0.2	ns	ns
Primorsky Kraj	2007–11	1,853	0.1	1,858	0.7	1,719	0.2	1,642	0.8	ns	ns
Amur Oblast	2007–11	216	0	198	0.4	148	0.1	195	1	ns	ns

Data from “Social-Hygienic Monitoring” system.

n=average number of samples per year.

ns=no samples analyzed.

nd=no data.

%=of samples exceeded threshold.

As for the percentage of samples which exceeded the Russian hygienic threshold (Tables IV and V), the nitrates dominate in all regions (from 1 to 14.4%); excesses of other pollutants in different regions are episodic and as a rule not high; aflatoxins reach 13% in Kamchatka; DDT reaches 9.2% (15.5% in local food) in Sakhalin; lead reaches 2.8% in Khanty-Mansi AO; cadmium reaches 1.8% in Komi; mercury reaches 1.7% in Yamalo-Nenets AO; and mercury in local fish reaches 9.3% in Yakutia.

In contrast to the knowledge about the chemical food contaminants data array, the Federal Automatic system “Social-Hygienic Monitoring” does not have any data on biological contaminants (specific contaminants) in the regions; therefore we could not evaluate the structure of biological contamination.

## Conclusion

The topic of food security in the non-aboriginal population of the Russia Arctic, Siberia and the Far East has been presented previously to some extent in Russian peer-reviewed journals: dietary imbalance and malnutrition, the predominance of carbohydrates, the shortage of milk products, vegetables, and fruits, deficits of vitamins and mineral nutrients in the diet, the abundance of microparasites (helminths) in local fish, and the chemical and infectious contamination of food. Until now, however, no unified indicators of food security for comparative assessment of the selected regions have been described.

This study presents the first complex comparative assessment of food security in 18 selected regions using unified food security indicators collected from statistical sources. The following indicators of food security have been analyzed: food costs (as a proportion of household income, %), food consumption (10 groups of products as a percentage of recommended quantities), and chemical and biological contaminants in food.

Food costs in the regions are high – from 23 to 43% of household income. Out of 10 groups of products, only 4 groups – fish products, cereals, sugar and plant oil – are consumed by the population of the selected regions in sufficient amounts. Meat consumption is very different, in some regions much lower than recommended quantities. There is a severely low consumption of milk products, eggs, vegetables, potatoes and fruits (and berries) in a majority of the selected regions. The deficit of fruits and berries is particularly alarming in a vast majority of the regions.

High levels of biological contamination of food (8–15% of samples exceeded the hygienic threshold) are registered in many regions. Chemical contamination generally is about half of the levels of biological contamination and fluctuated less (2–7%) in most regions. Chukotka food has appeared to be the worst in both biological and chemical contamination.

Ninety-four percent of food samples (all food in all regions) have been analyzed on 7 pollutants; 4 metals (lead, cadmium, arsenic and mercury), 1 product of mineral fertilizers (nitrates), 1 pesticide (DDT) and 1 mycotoxin (aflatoxin). Thus, of a high number of potential food pollutants, only 7 are under any real regular control, and among pesticides, only DDT is. According to the main Russian hygienic standard, the control of many contaminants (e.g. dioxins) in food is pursued only in cases of “reasonable assumptions about their possible presence in food.”

Total numbers of food samples analyzed in the selected regions, adjusted to the numbers of the population, constituted from 10 to 55 samples per 10,000, where Evenki AO (292) and Magadan Oblast (156) are the “leaders” in food contaminant laboratory control among the regions.

Nitrates, lead, cadmium and arsenic were assessed in all selected regions; mercury in local fish has not been analyzed in a majority of regions; DDT was out of control in 3 regions; aflatoxins have not been analyzed in 5 regions. Nitrates had the highest percentage of excess of the hygienic threshold in all regions; excesses of other pollutants in different regions were episodic and as a rule not high.

Final conclusion: improvement of the food supply and food accessibility in the regions of the Russian Arctic, Siberia and the Far East is of great importance. Both quantitative and qualitative control of chemical and biological contaminants in food is insufficient and demands radical enhancement aimed at improvement of food security. International studies based on these Russian food security indicators are also warranted.

## Footnotes

1 A set of submolecular, molecular, cellular and systemic changes that occur in the human body when exposed to environmental Arctic factors which promote serious changes in requirements and allocation of energy and nutrients in the organism.
